# Life cycle assessment (LCA) and energy assessment of the production and use of windows in residential buildings

**DOI:** 10.1038/s41598-023-47185-7

**Published:** 2023-11-13

**Authors:** Zbigniew Kowalczyk, Sebastian Twardowski, Mateusz Malinowski, Maciej Kuboń

**Affiliations:** Faculty of Production and Power Engineering, University of Agriculture in Cracow, Balicka 116B, 30-149 Cracow, Poland

**Keywords:** Environmental impact, Civil engineering

## Abstract

There is an observable scarcity of comprehensive research results comparing the environmental damage associated with both the production of windows and their subsequent operation. The environmental impact of the operation of windows depends on their thermal insulation parameters, and thus the amount of heat that must be generated to heat the building. The type of heating system and, above all, the type of fuel used to generate heat are also not without significance. Unfortunately, in Poland, a significant proportion of single-family houses operate on the fossil fuel heating system, including on coal and fuel oil. It is therefore important to present an environmental balance sheet of both the production and operation of windows for different variants of building heating. The purpose of the study was to determine: to what extent the manufacturing of windows of different construction and different insulation parameters affects the environment, to what extent does the negative environmental impact of the process of manufacturing with greater insulation compensate by the lower environmental impact related to savings on fuel (gas, coal, fuel oil) used to generate heat during the operation of windows. Three types of windows were selected for a detailed analysis: a triple-glazed aluminum construction, a double-glazed PVC construction and a triple-glazed PVC. The research results show that in the case of all impact categories, the greater environmental losses related to the improvement of the thermal insulation parameters of the windows at the production stage are fully compensated at the stage of their useful life, regardless of the type of fuel used to heat the buildings. Double-glazed PVC windows should be phased out of production due to significant environmental footprint associated with their operation.

## Introduction

Discussion on climate change, fossil fuel depletion, and energy security exacerbates the need to reduce energy consumption and CO_2_ emissions in the construction sector, which is responsible for a large percentage of the global environmental footprint^1^. In fact, buildings are large consumers of energy^2, 3^, materials and water, and important producers of waste and hazardous emissions^4^, including CO_2_^5^.

In Europe, the construction sector has significantly contributed to reducing greenhouse gas emissions, as it accounts for approximately 40% of total energy consumption^6, 7^. As the most energy-consuming sector in the United States, real estate accounts for 40.4% of the country’s total energy consumption and shows great potential for energy savings and emission reductions^8^. Improving the performance of buildings is key to achieving the Paris Agreement goal of reducing global warming by 1.5 °C^8^.

Windows are a structural element that affects the functioning of the building, including its energy efficiency and thermal and lighting comfort. They are also the main element that ensures contact between people and the environment^9^. Although windows must provide ventilation, soundproofing, wind and fire resistance, they are currently selected largely based on their thermal insulation properties^10^.

Window types and their characteristics have a major impact on eco- and cost effectiveness of buildings^11^. Next to renewable energy installations^12–14^ and warehouses^15–17^, replacing windows in buildings (especially in houses and public facilities) has become the main thermal renovation measure that reduces their energy intensity and ultimately improves their energy efficiency^18^. The window area in a building partition is approximately 20% of the total area of the building envelope. Total heat loss through windows is even more than 4 times higher than in the case of insulated building walls^19^. An important window parameter, the heat transfer coefficient, depends on the type of construction material. According to Saadatian et al.^20^, the value of the heat transfer coefficient has a large effect on smaller windows in warmer climates and on larger windows in colder climates. As mentioned above, the manufacturing of windows is a process that poses many threats to the natural environment. The life cycle of each window begins with the extraction of raw materials, which affects local ecosystems, depletes non-renewable resources, requires energy, and generates waste. Once extracted, the raw materials are transported to large manufacturing facilities where they are converted into standardized materials for use in window-specific secondary manufacturing. For windows as building elements, the impact of materials is greater, as they are typically high-value, technologically more advanced products than other building materials. This causes their disproportionate environmental impact compared to their mass and surface area^21^. The method chosen for the study is Life Cycle Assesment (LCA), which had been proven over several decades of use. The method is compliant with ISO 14000 standard, broadly used in scientific research. The LCA of windows is a very important tool for a comprehensive assessment of the impact of the production and operation of windows on the environment. This analysis takes into account not only the production process itself, but also the entire product life cycle, starting from the acquisition of raw materials, through the production process, the product’s use until disposal, the so-called “from cradle to grave”^22–26^. Scientific literature offers many articles, in which the LCA methodology was used to analyze the environmental impact of window manufacturing. Most of them investigated the impact of different materials used in the production of windows, as well as the impact of different production phases such as manufacturing, transport, installation and disposal^19^. Research on the impact of window production and on the environment using the LCA methodology was conducted, among others, by Refs.^27–34^. The production and use of windows as part of the life cycle of residential buildings was also considered in the environmental impact studies conducted by Refs.^35, 36^.

In recent years, optimizing energy use through efficient design has become an evolving research area^37^. The Home Energy Rating System (HERS) is one of the world’s leading initiatives integrating sustainable development features and energy saving measures^24, 38, 39^. The issue of energy consumption during the operation of windows was discussed, among others, by Refs.^37, 40–43^.

It can therefore be concluded that the purpose of LCA for windows is most often to compare the materials from which the frames are made, to determine the environmental impact of production, and to calculate heat savings during the operation of windows, especially the amount of heat required for the heating season^44, 45^.

Please note that windows with greater insulation can reduce the energy consumption needed to heat the building. In the long run, this decreases heating costs, and greenhouse gas emissions. At the same time, the manufacturing of windows with improved insulation usually requires more energy and increases greenhouse gas emissions. On the other hand, windows with less insulation can require less energy in the manufacturing process, but at the same time increase energy consumption during the building’s life cycle. This in turn leads to higher operational costs and increased environmental damage resulting from heating.

There is an observable scarcity of comprehensive research results comparing the environmental damage associated with both the production of windows and their subsequent operation. The environmental impact of the operation of windows depends on their thermal insulation parameters, and thus the amount of heat that must be generated to heat the building. The type of heating system and, above all, the type of fuel used to generate heat are also not without significance. Unfortunately, in Poland, a significant proportion of single-family residential buildings use heating systems that use fossil fuels, including coal and fuel oil. However, the process of replacing heating systems, forced, among other things, by adaptation to the recommendations of the so-called Green Deal, is a long-term process. It is therefore important to present an environmental balance sheet of both the production and operation of windows for different variants of building heating.

The selection and effects of window operation depend on many factors, including local conditions such as climatic factors, the location of the building in relation to the cardinal directions, the closest surroundings of the building, etc. Differences in temperature outside or inside the building or a different intensity of solar radiation in a given climatic zone completely change the amount of heat exchange through the windows. Therefore, the research results presented in the literature regarding the use of windows in a given region of the world cannot always be completely related to a building located in a different zone. The thermal performance of the same window systems can vary depending on many factors, including air infiltration and the dimensions and geometry of the windows. Therefore, there is still a need for in-depth research on heat transfer through windows and on the impact of window operation on the environment. In many cases, the available research results were published many years ago, which depreciates them due to technological progress. Moreover, the methodology used in individual studies of various authors is very different in terms of system boundaries and methods used, which means that they are not always comparable.

### Purpose and scope of research

There are many types of windows produced worldwide, with varied construction materials, structure itself, and production technology. In addition, windows are used in different climatic zones, where factors affecting their operation, such as temperature, sunlight, wind, etc., can be extremely different. The purpose of this work is to analyze the environmental impact of the production of various types of windows from the point of view of the environmental footprint associated with both the production of windows and their subsequent operation.

The research answers the following questions:

- what is the balance of environmental benefits of the production of windows with improved thermal insulation parameters?
- what are the relationships between the environmental footprint of the production of windows of various types and their subsequent operation?

The scope of work covered three types aluminum and PVC windows from one of the leading Polish manufacturers, with various types of glazing. The calculations and analyzes relate to 1 m^2^ of the window area.

Selecting windows with the lowest environmental footprint in their entire life cycle as early as the design phase is important, to minimize the environmental impact of buildings^46^. The results of the research can therefore be used to support early decisions of designers regarding the selection of window materials and components from the point of view of ultimate environmental benefits related not only to the manufacturing process, but also to the heating of buildings in given climatic conditions. The results will also allow predicting differences in the energy consumption needed to heat buildings depending on the types of windows used.

## Materials and methods

### Subject of research

The subject of the research are three types of windows:

- triple-glazed aluminum window;
- double-glazed PVC window;
- triple-glazed PVC window.

**Figure 1:**
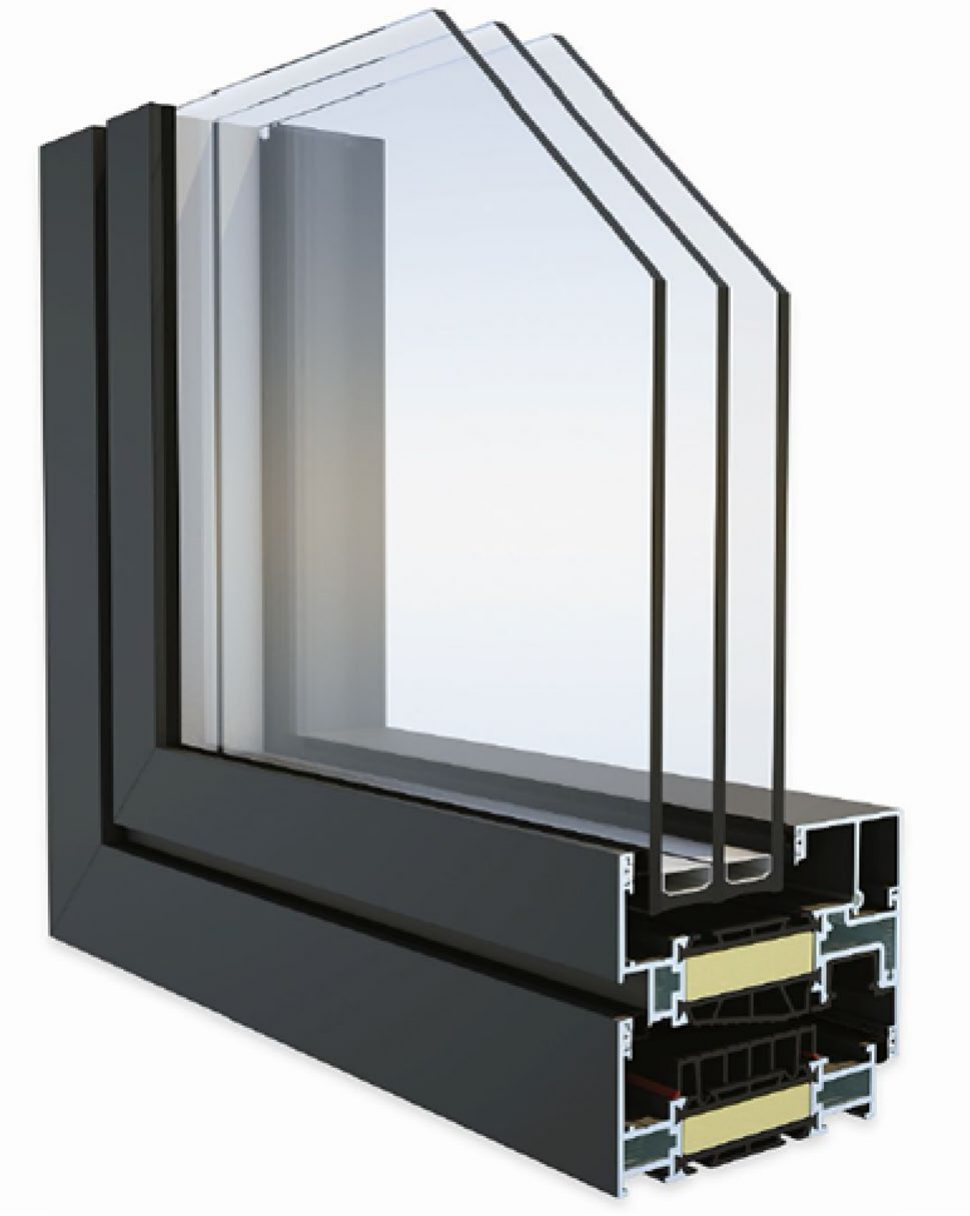
Cross-section of a 1000 × 1000 mm aluminum window with 4/18/4/18/4 unit glass.

**Figure 2:**
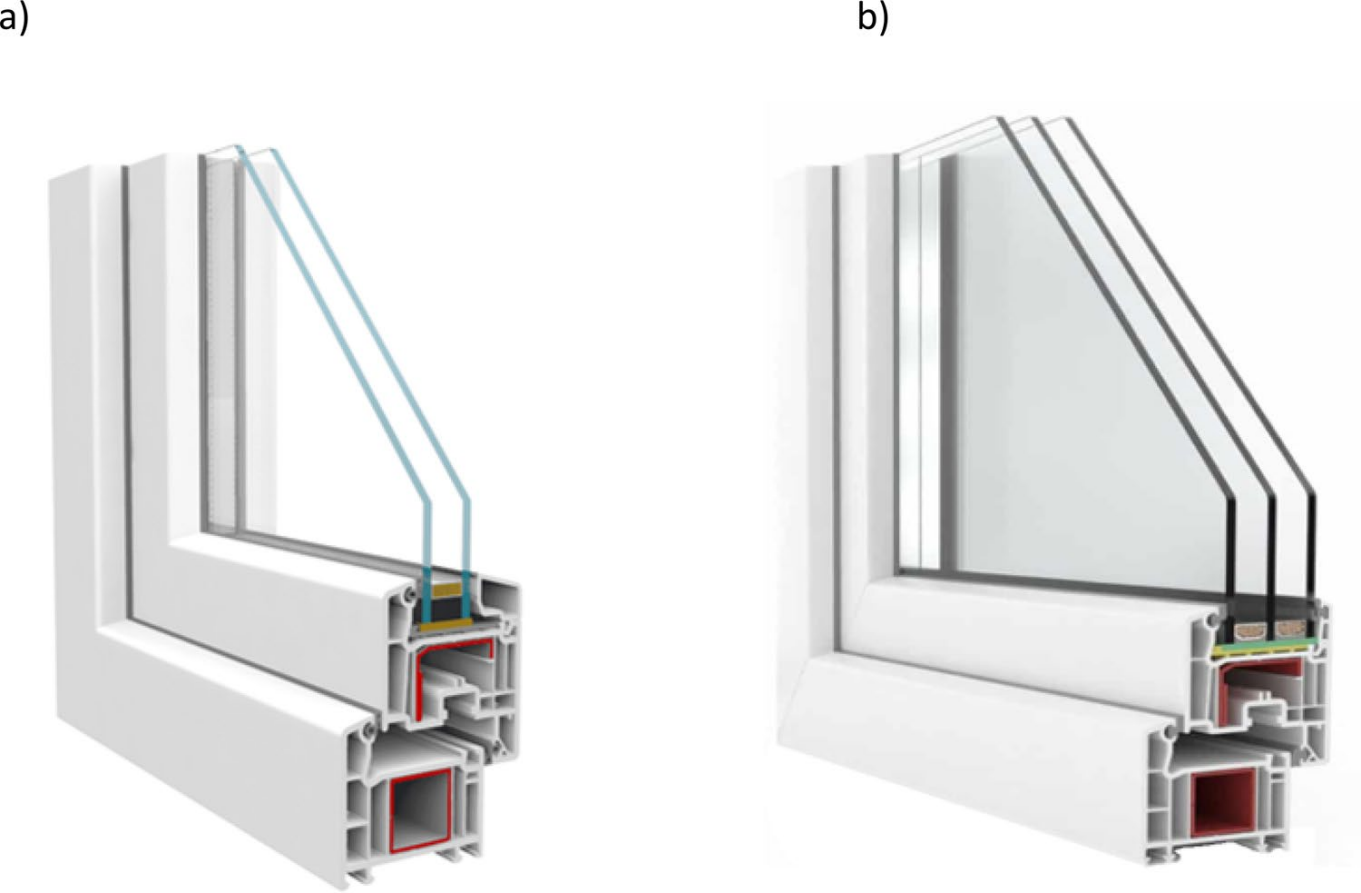
Cross-section of a 1000 × 1000 mm PVC window: (**a**) with 4/14/4 unit glass, (**b**) with 4/14/4/14/4 unit glass.

Figures 1 and 2 show the structure of the tested windows.

The aluminum window, the cross-section of which is shown in Fig. 1, has the lowest heat transfer coefficient, at U_w_ = 0.82 W·m^−2^·K^−1^. It is made of aluminum profiles with three insulating chambers and gaskets, additional thermal insulating pads, as well as insulation foam in the sash frame and in the casing. It has glazing made of three 4 mm thick panes separated by an 18 mm wide aluminum frame, which form two chambers additionally supplemented with argon gas.

A PVC window with single-chamber (double-pane) unit glass, the cross-section of which is shown in Fig. 2a, has the highest heat transfer coefficient, at U_w_ = 1.10 W·m^−2^·K^−1^. The construction of the window consists of PVC profiles with five insulating chambers, with gaskets and reinforcements made of steel profiles. The glazing consists of two 4 mm thick panes separated by a 14 mm wide aluminum frame, which form one chamber, additionally filled with argon.

The last window, the cross-section of which is shown in Fig. 2b, is a PVC window with double-chamber (three-pane) unit glass. This window has a heat factor at U_w_ = 0.92 W·m^−2^·K^−1^ and is made of PVC profiles with five insulating chambers, with gaskets and reinforcements made of steel profiles. Its glazing consists of three panes, 4 mm thick, separated by 14 mm wide aluminum frames, which create two chambers additionally filled with argon. A summary of the most important window parameters is shown in Table 1.

**Table 1:** General characteristics of windows.

Window type	Heat transfer coefficient U_w_ (W·m^−2^·K^−1^)	No. of isolating chambers	Service life (years)
Triple-glazed aluminum window	0.82	3	50
Double-glazed PVC window	1.10	5	25
Triple-glazed PVC window	0.92	5	25

### System boundaries

**Figure 3:**
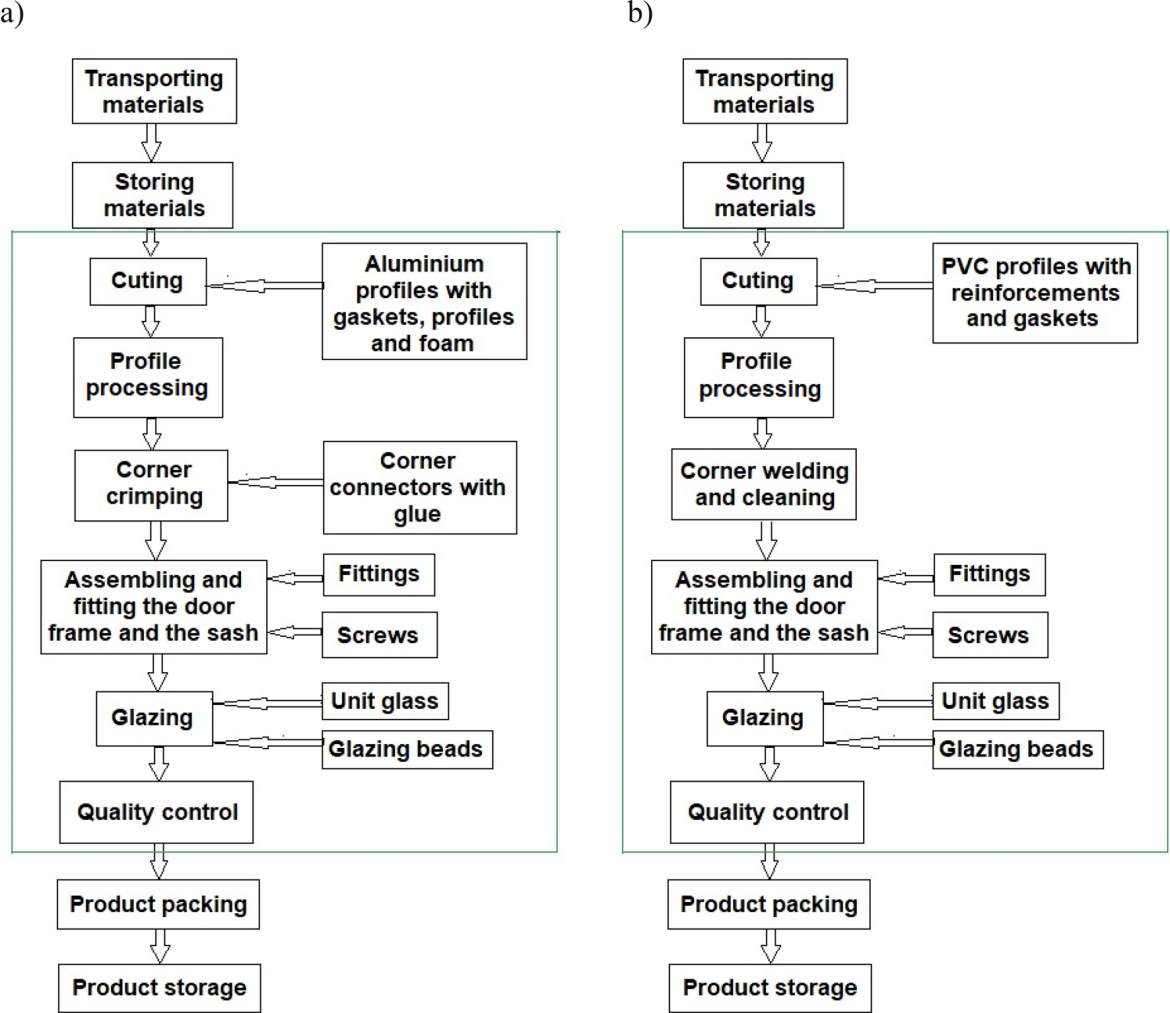
The limits of the production system for the windows: (**a**) aluminum, (**b**) PVC.

The study used a “cradle-to-gate” approach. Figure 3 shows the system boundaries for the manufacturing of the three studied types of windows.

Both manufacturing processes include suitable operations, from profile cutting to quality control. Earlier and further operations, i.e. those related to the delivery of manufacturing materials, as well as packaging, storage, external transport and sales, have not been taken into account. Unfortunately, the above-mentioned stages are carried out in a very different way, which makes it impossible to calculate their LCA. The process of packing windows depends on the customer’s requirements, the distance, and the number of ordered windows. Sometimes windows are not packed at all. The same is true of storage, the period of which varies greatly, and sometimes windows are not stored. Both packaging and storage, due to the technologies used, do not significantly affect the environmental impact of the production process. As for the transportation of production materials, they are provided by other external companies that cooperate with the window manufacturer. Transportation is combined and together materials are transported for the production of the windows included in the analysis, but also for the production of doors and other types of manufactured windows. The above hampers the analysis process.

### Impact assessment methodology

The life cycle assessment guidelines are regulated by the ISO 14000 standard series (from 14040 to 14049) and their Polish equivalents. They include the requirements and rules for performing the LCA analysis, as well as the rules for interpreting its results. The LCA method is defined by the ISO 14040 standard as a method to assess the impact of production inputs on the natural environment.

According to the ISO 14040 standard series, LCA testing methodology includes four phases^47^.

- goal and scope definition;
- life cycle inventory analysis (LCI);
- life cycle impact assessment (LCIA);
- life cycle Interpretation.

To determine the environmental dependencies of all inputs and outputs covered by the scope of LCA, and to estimate their impact on the environment, 8.1.0.60 SimaPro software was used, which, according to Bahramian^48^, was used in approximately 40% of LCA studies in the construction sector. The adopted functional unit was 1 m^2^ of the window surface, as in research of Intini et al.^49^. The applied environmental impact assessment methods were: ReCiPe MidPoint and ReCiPe Endpoint. Endpoint indicators show the environmental impact at three higher levels of aggregation: (1) the effect on human health, (2) biodiversity, ecosystem, and (3) resource scarcity. Indicators in individual impact categories for the MidPoint and EndPoint methods are presented in Table 2. Midpoint indicators are intermediate measures of environmental impact that reflect changes in the natural environment caused by emissions or resource use. For example, greenhouse gas emissions can be expressed as carbon dioxide equivalents (CO2), which indicate the global warming potential of different gases. Midpoint indicators are often easier to calculate and understand than endpoint indicators, as they are closer to the source of the impact and less affected by uncertainties and assumptions. However, midpoint indicators could not capture the full consequences of environmental changes for human well-being or ecosystem services, and could not reflect the relative importance or severity of different impact categories.

**Table 2:** ReCiPe endpoint and midpoint impact categories^50^.

Endpoint		Midpoint	
Impact categories	Abbreviation	Impact categories	Abbreviation
Human health		Climate change	CC
Climate change human health	CCHH	Ozone depletion	OD
Ozone depletion	OD	Terrestrial acidification	TA
Human toxicity	HT	Freshwater eutrophication	FE
Photochemical oxidant formation	POF	Marine eutrophication	ME
Particulate matter formation	PMF	Human toxicity	HT
Ionizing radiation	IR	Photochemical oxidant formation	POF
Ecosystems		Particulate matter formation	PMF
Climate change ecosystems	CCE	Terrestrial ecotoxicity	TET
Terrestrial acidification	TA	Freshwater ecotoxicity	FET
Freshwater eutrophication	FE	Marine ecotoxicity	MET
Terrestrial ecotoxicity	TET	Ionizing radiation	IR
Freshwater ecotoxicity	FET	Agricultural land occupation	ALO
Marine ecotoxicity	MET	Urban land occupation	ULO
Agricultural land occupation	ALO	Natural land transformation	NLT
Urban land occupation	ULO	Water depletion	WD
Natural land transformation	NLT	Mineral resource depletion	MRD
Resources		Fossil fuel depletion	FD
Metal depletion	MD		
Fossil depletion	FD		

The Endpoint characterization factors used in ReCiPe can be described as follows: Human Health, Ecosystems, and Resources. Human Health, expressed as the number of years of life lost and the number of years of life with a disability. These are combined as disability adjusted life years (DALYs), and the unit is years. Ecosystems are expressed as the loss of species over a certain area, during a time period. The unit is years. Resource scarcity, expressed as the surplus costs of future resource production over an infinite time frame (assuming constant annual production), considering a 3% discount rate. The unit is USD^51^. Endpoint indicators are often more relevant and comprehensive than midpoint indicators, as they show the ultimate outcomes of environmental changes and allow for a more integrated and consistent comparison of different impact categories. The main advantage of the ReCiPe method is that it transforms a long list of life cycle inventory results into a limited number of indicator results. These indicator scores express relative severity in terms of environmental impact. ReCiPe is unique in that it provides both a midpoint- and an endpoint approach, which is a strong suit in that it may be relevant for a wider spread of uses than the methods only taking one of the mentioned approaches into consideration; specific data and statistics can be extracted from the midpoint level, while the endpoint level may provide information that is easier to understand and interpret.

### Life cycle inventory (LCI)

The life cycle inventory (LCI) allows to identify the resources used for manufacturing, and resulting emissions, which are necessary for the life cycle assessment, i.e. the determination of the product’s potential effects on human health and its environmental footprint^52^.

Tables 3 and 4 present the individual components of the tested windows, as well as their material type and weight.

**Table 3:** The weight of materials of a 1000 × 1000 mm aluminum window.

Element	Type of material	Weight (kg)
Unit glass	Glass	22.52
Sash frame, casing, glazing beads, accessories, spacers	Aluminum alloy	9.97
Thermal pads, central profile	Artificial integrated ABS copolymer	2.26
Fitting	Galvanized steel	2.02
Thermal inserts	Polyurethane foam	0.60
Gaskets	Thermoplastic polyester elastomer (TPE)	0.40
Reinforcement, accessories	PVC	0.39
Screws	Stainless steel	0.20

**Table 4:** The weight of 1000 × 1000 mm PVC window materials.

Element	Type of material	Weight (kg)	
		Double-glazed window	Triple-glazed window
Unit glass	Glass	13.07	19.61
Frame, sash, glazing beads, accessories	PVC	12.44	12.44
Reinforcement, fittings	Galvanized steel	7.98	7.98
Gaskets	Thermoplastic polyester elastomer (PTE)	0.40	0.40
Spacer	Aluminum alloy	0.33	0.66
Screws	Stainless steel	0.20	0.20

The production technology of aluminum windows begins with cutting aluminum profiles to size using mechanical saws. Then the profiles are properly processed on the milling machines. The corners of the profiles are then joined with corner connectors, filled with a special glue, and assembled on a pneumatic crimping machine. The frame and leaf, crimped separately, are placed on special stands until the glue dries. The next stage of window production is fitting, i.e. equipping the sash and frame with a set of elements that enable opening of the window: a handle, hinges, envelope strips and catches, and assembling glass. Next, the window is subjected to quality control.

The technological process for the production of double and triple-glazed PVC windows is the same and the only difference in the process is that different units of glass panes and glazing beads are installed. PVC profiles are cut to size using mechanical saws, then milled, welded, and cleaned. Next, sashes and window frames are assembled and glass panes, glazing beads, handles, hinges, envelope strips, and catches are installed. Then the window is subjected to quality control.

### Environmental benefits from the use of the analyzed windows

Heat conducted through building partitions, including windows, is expressed by the thermal transmittance value (U_w_ = W·m^−2^·K^−1^). A lower U_w_ value means higher resistance to heat flow and better insulation. The total U_w_ value for a window is measured from the combined effect of glass, frame, air seals, and the spacer between panels. This parameter is the basis for calculating the environmental benefits of using windows with different thermal insulation properties.

To calculate the environmental impact of the use of the analyzed windows, a standard residential building was used, located in the third climatic zone of Poland, where the average annual temperature is 7.6 °C^53^. The facility area is 169 m^2^, its cubic capacity is 579 m^3^, and the glazing area is 41 m^2^. The exterior walls of the building are insulated with a 10 cm polystyrene insulating layer. Its 40° slope roof has a 10 cm layer of mineral wool. The temperature inside the building is 20 °C. The buildings do not have a usable attic or garage. Ventilation is natural, without a cooling system. The maximum required heat transfer coefficients through the external partitions of the test object are as follows^54^:

- for walls: 0.25 W·m^−2^·K^−1^,
- for the roof: 0.20 W·m^−2^·K^−1^,
- for floors (over unheated basement): 0.25 W·m^−2^·K^−1^.

According to Szul^55^, the average heat consumption to heat buildings located in the climate zone mentioned above is approximately 120 kWh·m^−2^·year^−1^. The value of the heat transfer coefficient through the windows depended on the type of window adopted.

The heat demand resulting from the use of windows with different U coefficients (option 0—triple-glazed aluminum window, option 1—double-glazed PVC window, and variant 2—triple-glazed PVC window) was calculated using Audytor OZC 7.0 software (SANKOM, Poland). The adopted solar transmittance coefficient of the windows was 0.5. Heat demand was related to the functional unit, that is, 1 m^2^ of windows, to indicate differences in environmental impact between variant 0 (with the best thermal insulation parameters) and the other variants analyzed (windows with a PVC frame—double and triple glazing). The differences in heat demand were also converted into a reduction in the wear of specific energy carriers during the adopted service life of the windows, that is, 25 years, which is the lowest durability of windows (PVC). When preparing the environmental balance sheet for the production and useful life of windows, it was assumed that the life of windows with a PVC frame is 25 years and that of an aluminum window 50 years (as declared by the manufacturer). The research included the following energy carriers, as they were the most popular heat sources in Polish households:

- hard coal (average calorific value of 28.9 MJ·kg^−1^, density of 800 kg·m^−3^^56^, with 70% efficiency of the heating system),
- natural gas (average calorific value of 35.5 MJ·kg^−1^, density 0.78 kg·m^−3^^57^, with 90% efficiency of the heating system),
- fuel oil (average calorific value of 42.6 MJ·kg^−1^, density 860 kg·m^−3^^58^, with 90% efficiency of the heating system).

Then, according to the methodology described in the chapter “Impact assessment methodology” section, the environmental impact of reducing the heat demand in the analyzed buildings was determined, taking into account various types of windows within the adopted period of their useful life. The effect of window aging and the resulting increase in U-value was not analyzed. The results of environmental analyzes were presented as a comparison of environmental loads between the window model with the best thermal insulation parameters (with an aluminum frame) and other models, in individual impact categories (these differences in environmental loads result from the extraction of conventional fuels and the production of heat from them).

## Results and discussion

### Characterization results with ReCiPe midpoint and endpoint

Table 5 presents the results of the environmental impact analysis carried out using the ReCiPe MidPoint method.

**Table 5:** Results of the ReCiPe Midpoint analysis for the production of the three tested window models.

Impact category	Unit	Triple-glazed aluminum window	Double-glazed PVC window	Triple-glazed PVC window
CC	kg CO_2_-eq	111.61	63.20	73.13
OD	kg CFC-11-eq	0.000009	0.000003	0.000004
TA	kg SO_2_-eq	0.7619	0.3081	0.3909
FE	kg P-eq	0.0321	0.0128	0.0147
ME	kg N-eq	0.0256	0.0135	0.0162
HT	kg 1.4 DB-eq	37.98	14.03	16.66
POF	kg NMVOC	0.4672	0.3159	0.3644
PMF	kg PM_10_-eq	0.3064	0.1461	0.1736
TET	kg 1.4-DB-eq	0.0068	0.0038	0.0045
FET	kg 1.4-DB-eq	25.25	1.30	2.18
MET	kg 1.4-DB-eq	21.76	1.17	1.93
IR	kBq U235-eq	5.49	2.21	2.84
ALO	m^2^ yr	2.97	1.31	1.74
ULO	m^2^ yr	1.127	0.569	0.684
NLT	m^2^	0.0148	0.0067	0.0087
WD	m^3^	1.17	2.67	2.75
MD	kg Fe-eq	25.06	17.43	19.52
FD	kg oil-eq	30.59	23.49	26.07

The adopted functional unit was the window area, i.e. 1 m^2^. The analysis covered 18 environmental indicators, the designation of which is presented in Table 2. Generally speaking, production of aluminum windows with the best thermal insulation parameters (thermal transfer coefficient U_w_ = 0.82 W·m^−2^·K^−1^, is also characterized by the greatest negative impact on the environment, as evidenced by the values of MidPoint indicators in all impact categories. For most indicators, the environmental impact of this model is 1.3–3.0 times greater compared to a double-glazed PVC window, which by the worst thermal insulation parameters (U_w_ = 1.10 W·m^−2^·K^−1^). The values of two of the indicators, that is, freshwater ecotoxicity (FET) and marine ecotoxicity (MET), are approximately 19 times higher in the manufacturing of an aluminum window compared to a PVC window because of the use of aluminum as a construction material. The only exception in the MidPoint analysis is the Water Depletion Index (WD), which, unlike the others, is more than twice as high for the manufacturing of PVC windows compared to aluminum windows. This, in turn, is due to the higher weight of PVC material in the design of PVC windows. When comparing the environmental impact of PVC windows: triple-glazed and double-glazed, 10–30% differences in MidPoint indicators can be observed. This indicates a slightly greater environmental impact of triple-glazed windows. Much greater differences, approximately 70%, were recorded in terms of freshwater ecotoxicity (FET) and marine ecotoxicity (MET), which is mainly due to the much greater weight of aluminum elements in triple-glazed windows (Table 4).

When analyzing one of the most important indicators in Table 4, i.e., climate change, note the nearly double difference in CO_2_ emissions between a double-glazed PVC window and an aluminum one. This is because windows have a huge impact on the environmental impact in this category on the entire building. According to Radhi and Sharples^59^, each square meter of glazing can increase the total CO_2_ emission by almost 30% compared to the same opaque wall surfaces. However, it should be emphasized that the LCA results are difficult to compare due to the lack of international standardization of the applied methodologies. Therefore, the application of the LCA methodology in construction is still a major challenge and a still valid research area^4^.

Table 6 presents the results of the environmental impact analysis carried out using the ReCiPe EndPoint method.

**Table 6:** ReCiPe endpoint analysis results for the manufacturing of the three window types tested.

Impact category	Unit	Triple-glazed aluminum window	Double-glazed PVC window	Triple-glazed PVC window
CCHH	DALY	0.0001562529	0.0000884883	0.0001023908
OD	DALY	0.0000000163	0.0000000084	0.0000000105
HT	DALY	0.0000265795	0.0000098215	0.0000116626
POF	DALY	0.0000000182	0.0000000123	0.0000000142
PMF	DALY	0.0000796876	0.0000380058	0.0000451445
IR	DALY	0.0000000901	0.0000000364	0.0000000467
Total human health	DALY	0.000263	0.000136	0.000159
CCE	species.yr	0.0000008850	0.0000005012	0.0000005799
TA	species.yr	0.0000000044	0.0000000018	0.0000000023
FE	species.yr	0.0000000014	0.0000000006	0.0000000007
TET	species.yr	0.0000000010	0.0000000006	0.0000000007
FET	species.yr	0.0000000216	0.0000000011	0.0000000019
MET	species.yr	0.0000000038	0.0000000002	0.0000000003
ALO	species.yr	0.0000000357	0.0000000158	0.0000000209
ULO	species.yr	0.0000000233	0.0000000118	0.0000000142
NLT	species.yr	0.0000000257	0.0000000114	0.0000000147
Total ecosystems	species.yr	0.000001002	0.000000544	0.000000636
MD	$	1.79	1.25	1.40
FD	$	5.06	3.88	4.31
Total resources	$	6.85	5.13	5.71

In total, 17 environmental indicators were analyzed, the designation of which is presented in Table 2. Considering the total environmental impact of window production in the Human health category, it can be observed that the production of a window with the best thermal insulation parameters, i.e. the aluminum window, has almost double negative impact on the environment as compared to the production of a double-glazed PVC window, i.e. with the highest heat transfer coefficient. Citherlet et al.^60^ point out, however, that even if advanced windows have a slightly higher environmental impact during their life cycle, the difference is not significant compared to the energy gains they provide during usage thanks to their insulating properties. When comparing the environmental impact of double and triple glazed PVC windows in the human health category, the difference is much lower and amounts to approximately 20%. In the Ecosystems impact category, similar relationships were observed, but the differences between the aluminum window and the double-glazed PVC were slightly smaller, while in the case of both types of PVC windows, the same differences in environmental impact were found as in the Human health category. Similar relationships, but at an even lower level, were observed in the case of the third category of environmental impact, i.e., Resources. The production of an aluminum window had approximately 30% more negative impact on the depletion of natural resources compared to a double-glazed PVC window, while the difference between PVC windows was only approximately 10%. The significant environmental footprint associated with the production of aluminum windows compared to PVC windows can be caused, for example, by: high energy consumption in the manufacturing process, as indicated by Cabeza et al.^61^. Research by Asif et al.^62^, proves that in the case of an aluminum window, the production of a 1.2 m × 1.2 m window frame requires 6 GJ of energy, while for a PVC window, only 2.9 GJ.

### Results of the life cycle impact assessment (LCIA)

**Figure 4:**
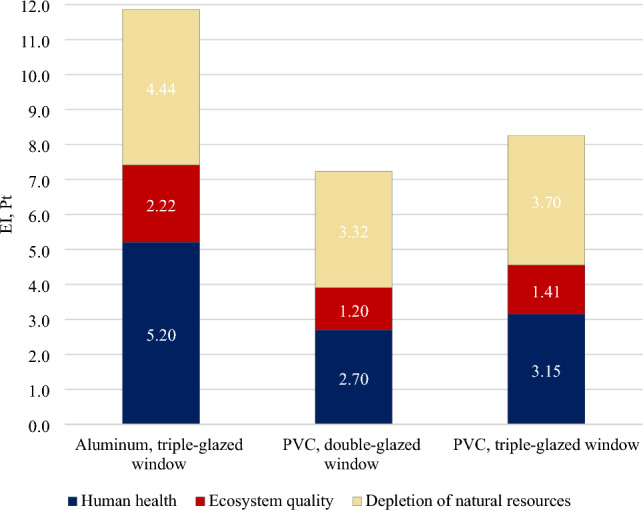
Environmental impact (EI) of window production in three categories of damage.

The impact assessment is the third stage of the life cycle assessment, and it is used in accordance with the ISO 14044 series^63^. Figure 4 shows the weighted environmental impact EI for the manufacturing of the three window types studied.

Upon analyzing the size of the environmental impact shown in Fig. 4, it can be observed that generally the lowest environmental impact (EI = 7.22 Pt) is characteristic of double-glazed PVC windows, which have the worst thermal insulation properties. Improving the thermal insulation properties of PVC windows by increasing the number of panes from two to three increases the environmental impact related to manufacturing by approximately 14% (EI = 8.26 Pt). Horup et al.^64^ point out that, based on a comparison of double- and triple-glazed windows, the expected net energy savings achieved in their use phase can be compromised by the relatively greater environmental impact exerted during the production phase. The advantage of PVC as a window frame construction material is also the fact that it can be combined with many additives to achieve a broad range of properties, from rigid plastics to flexible materials. It is the above characteristics, as well as the mechanical strength and chemical resistance, that make PVC so popular in the production of window frames^65^.

Although the thermal insulation parameters of aluminum windows are better than the two tested types of PVC window, they require completely different construction materials and production technology. Furthermore, aluminum windows have a negative environmental impact: The EI value is 11.86 Pt, which is more than 64% higher than in the case of a double-glazed PVC window and more than 43% higher than in the case of a triple-glazed PVC window. The above results are confirmed by research by Sinha and Kutnar^66^, which shows that aluminum frames cause the greatest burden on the environment. According to Asif^62^, the cause of environmental hazards associated with the production of aluminum windows could be the release of hazardous pollutants and the high energy consumption during the processing of aluminum. The same research shows that the production of PVC windows is slightly less harmful, but also releases large amounts of pollutants. On the other hand, wooden window frames have the lowest environmental impact, which is also confirmed by research, for example, Stachowiak-Wencek et al.^67^.

Also, Tushar et al.^68^ conducted a comparative analysis of windows with different frames which showed the superiority of PVC windows compared to aluminum. This is mainly due to a lower environmental impact, recyclability, and lower energy consumption in the production phase. As much as 44% of the total environmental impact related to the production of aluminum windows is related to the depletion of natural resources. In the case of PVC windows, the Human health category dominates in the structure of environmental impact, accounting for approximately 45–46% of the EI index. The production of all three types of windows has the least impact on the environment in the Ecosystems category. Interestingly, there were no significant differences between the three window types covered by the study, as was the case with the other two categories, namely: Human Health and Resources, where the value of the EI index ranges from 17 to 19% of the total environmental impact.

### Analysis of the environmental impact related to the use of windows and the environmental balance

Table 7 summarizes the results of the calculations regarding the change in heat demand as a result of the use of the windows analyzed per 1 m^2^ window in relation to variant 0 (aluminum window with the best thermal insulation parameters). It also presents a reduction in the demand for various types of fuel. Their characteristics are given in “Characterization results with ReCiPe midpoint and endpoint” section (in relation to 1 year of useful life and the entire 25-year period of operation).

**Table 7:** Change in the demand for heat and energy carriers depending on the type of window used.

Type	Double-glazed PVC window	Triple-glazed PVC window
Change in heat demand in a building (kWh·year^−1^) in relation to variant 0	796.1	284.2
Change in heat demand per 1 m^2^ windows (kWh·year^−1^) in relation to variant 0	19.4	6.9
Hard coal demand (kg·year^−1^) in relation to variant 0	4.16	1.48
Natural gas demand (m^3^·year^−1^) in relation to variant 0	1.70	0.60
Heating oil demand (kg·year^−1^) in relation to variant 0	1.81	0.64

In the model building, the heat demand was 19,075.2 kWh·year^−1^. Of all the types of windows analyzed, by far the smallest building heat demand can be obtained (Table 7) for windows in variant 0 (triple-glazed, with an aluminum frame). Their heat transfer coefficient is the lowest, and therefore they are a good insulator. Based on 1 m^2^ windows, they allow saving respectively 19.4 kWh of heat per year in relation to double-glazed windows with a PVC frame and 6.9 kWh of heat in relation to triple-glazed windows with a PVC frame. The above heat savings reduce the demand for the energy carriers necessary to generate the amount of heat necessary to heat the building. The least favorable in this regard are double-glazed windows with a PVC frame. In that case, the heat consumption in the building is greater over the 25 years of useful life. It would require 104 kg of coal or 42.5 m^3^ gas or 45.2 kg of heating oil more compared to windows with an aluminum frame. In the case of using triple-glazed PVC windows, the difference in consumption will be as follows: 37 kg of coal, 15 m^3^ natural gas, and 16 kg of heating oil more are required compared to the operation of windows with an aluminum frame. Please note that according to Zhou et al.^69^, windows can contribute to 30% heat losses in the house in winter and even up to 40% according to Gramlick^70^. Therefore, the above differences are of great importance in the total heating balance of the building.

One of the objectives of the analysis was to answer the question whether the environmental footprint of aluminum window manufacturing (which is greater than in PVC windows) can be offset by a lower demand for heat during its useful life in a typical residential building located in the third climatic zone in Poland. According to Buyle and Braet^71^ in standard buildings, it is the useful life phase that accounts for up to 90% of the total environmental footprint, mainly due to heating and/or cooling.

Table 8 shows the result of the comparison of the environmental impact (resulting from the reduction of energy carriers used to heat the building) of aluminum and other types of windows over a 25-year service life period (Midpont ReCiPe model). Positive values in all impact categories mean that the useful life of windows with a PVC frame has a more negative impact on the environment than that of windows with an aluminum frame. The use of windows with an aluminum frame (variant 0) is more environmentally friendly, as it saves energy. The greatest environmental footprint is that of double-glazed PVC windows (regardless of the energy carrier used).

**Table 8:** Comparison of the environmental impact of aluminum and PVC windows in terms of reducing heat demand; a ReCiPe—MidPoint analysis.

Impact category	Unit	Energy carrier/variant of the analyzed windows					
		Coal		Natural gas		Heating oil	
		Double-glazed PVC window	Triple-glazed PVC window	Double-glazed PVC window	Triple-glazed PVC window	Double-glazed PVC window	Triple-glazed PVC window
CC	kg CO_2_-eq	343.51	122.18	133.83	47.57	183.40	65.18
OD	kg CFC-11-eq	0.000013	0.000005	0.000010	0.000004	0.000059	0.000021
TA	kg SO_2_-eq	1.6913	0.6016	0.6847	0.2427	0.5809	0.2061
FE	kg P-eq	0.0187	0.0067	0.0008	0.0003	0.0020	0.0007
ME	kg N-eq	0.0161	0.0057	0.0042	0.0015	0.0109	0.0039
HT	kg 1.4 DB-eq	18.9376	6.7356	12.5828	4.4593	6.0124	2.1345
POF	kg NMVOC	1.9066	0.6781	0.2303	0.0817	0.3898	0.1383
PMF	kg PM_10_-eq	0.7140	0.2540	0.1565	0.0555	0.1713	0.0608
TET	kg 1.4-DB-eq	0.0077	0.0027	0.0126	0.0045	0.0059	0.0020
FET	kg 1.4-DB-eq	0.0269	0.0096	0.8325	0.2950	0.1426	0.0506
MET	kg 1.4-DB-eq	0.0764	0.0272	0.2544	0.0902	0.1153	0.0409
IR	kBq U235-eq	1.1337	0.4033	0.7624	0.2708	20.9055	7.4131
ALO	m^2^ yr	7.1221	2.5335	0.3052	0.1084	0.7935	0.2816
ULO	m^2^ yr	3.9871	1.4182	0.1166	0.0414	0.5473	0.1942
NLT	m^2^	0.0248	0.0088	0.0266	0.0094	0.1153	0.0409
WD	m^3^	0.2379	0.0846	0.0678	0.0241	0.5753	0.2040
MD	kg Fe-eq	2.4680	0.8779	1.8056	0.6414	4.3423	1.5428
FD	kg oil-eq	123.15	43.81	80.19	28.41	112.28	39.81

As mentioned above, when calculating the environmental load related to the operation of windows, a 25-year useful life period was adopted, which is consistent with the data provided in the literature^11, 62, 72^. When examining the results of the environmental analysis of the 25-year useful life phase (Table 8), a clear pattern emerges. These results contrast with those of the window production process (Table 5). It becomes evident that the environmental advantages from reduced fossil fuel demand do not cover all impact categories. Specifically, they do not offset the adverse environmental effects of producing aluminum windows. As a result of the analysis in the Midpoint ReCiPe model, it was found that for indexes such as: Freshwater eutrophication (FE), Human toxicity (HT), Freshwater ecotoxicity (FET), Marine ecotoxicity (MET), Ionizing radiation (IR), and Metal depletion (MD) The differences in environmental impact during the operational phase are too small to offset the negative environmental impact generated in the manufacturing phase.

For example, considering the category Climate change (CC), the manufacturing of aluminum windows generates 48.4 kg of CO_2_-eq more than that of double-glazed PVC windows, but the environmental benefits (reduced footprint) due to the reduction of coal mining and burning for heating purposes in the 25-year useful life of these windows amount to as much as 343.5 kg of CO_2_-eq. In the case of the climate change (CC) index, as well as Terrestrial ecotoxicity (TET), Natural land transformation (NLT), Fossil depletion (FD) and Water depletion (WD), the environmental impact resulting from the reduction of demand for all analyzed energy carriers (coal, gas and oil) fully covers (compensates for) the negative environmental impact associated with the production of aluminum windows.

The ReCiPe EndPoint analysis of the weighted environmental impact (EI), the reduction of heat demand in the building as a result of the use of various windows throughout their useful life, showed that installing windows with aluminum frames with a U_w_ = 0.82 W·m^−2^·K^−1^ heat transfer coefficient brings great environmental benefits compared to windows with a PVC frame. If the building is heated with hard coal, the positive environmental effect of installing an aluminum frame window is EI = 66.57 Pt in relation to a double-glazed PVC window, while in relation to a triple-glazed PVC window it is EI = 23.68 Pt. If the building is heated with natural gas, the positive environmental effect of using an aluminum window compared to a PVC window is as follows: EI = 31.66 Pt for a double-glazed window and EI = 11.23 Pt for a triple-glazed window. In a building heated with heating oil, the positive effect of using a window with an aluminum frame compared to a PVC window was the following. EI = 44.01 Pt for a double-glazed window and EI = 15.62 Pt for a triple-glazed window.

**Figure 5:**
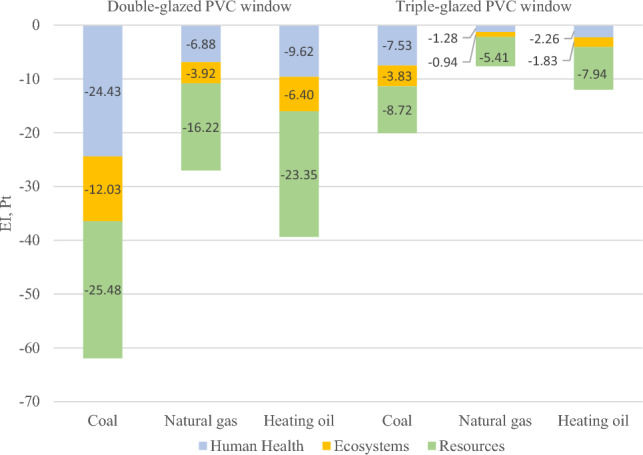
Environmental balance of manufacturing and operation of PVC windows compared to aluminum windows.

According to Souviron et al.^11^ during the LCA of window manufacturing, greater use of resources during production to improve the thermal insulation characteristics related to the useful life of windows is justified. Figure 5 shows the total environmental balance of the manufacturing phase and the useful life of PVC windows compared to aluminum windows for 3 different energy carriers used to heat the building. The negative impact of the aluminum window manufacturing process has been balanced for each of the energy carriers. Taking into account the entire useful life period of an aluminum window (50 years) and PVC (25 years), an aluminum window will have a positive environmental impact in all damage categories analyzed compared to a PVC window. When analyzing Fig. 5, it can be seen that the production and operation of PVC double-glazed windows increases the negative environmental impact compared to the production and operation of aluminum windows by, respectively: 61.9 Pt—in the case of coal-fired heating systems, 27.0 Pt—when using gas and 39.3 Pt—when using fuel oil. The overall balance for triple-glazed PVC windows is slightly more favorable. The production and operation of triple-glazed PVC windows compared to aluminum windows results in environmental losses of: 20.0 Pt (coal-fired furnaces), 7.6 Pt (gas-fired furnaces) and 12.0 Pt (fuel oil-fired furnaces).

## Conclusions

The purpose of this work was to analyze an environmental impact of the manufacturing of three types of windows, with different thermal insulation parameters. The study related to the production stage, and the service life. The research results show that in the case of all impact categories, the greater environmental losses related to the improvement of the thermal insulation parameters of the windows at the production stage are fully compensated at the stage of their useful life, regardless of the type of fuel used to heat the buildings.

The environmental benefits achieved by using aluminum windows over double-glazed PVC in buildings range from 27.0 to 61.9 Pt, depending on the heating system. The most significant environmental savings from installing aluminum windows are observed in buildings with coal-fired furnaces. Given the exceptionally high prevalence of coal-fired stoves in Poland (exceeding 50%), it’s recommended that PVC double-glazed windows be phased out of production. Concurrently, modernizing the heating sector—shifting away from coal-fired stoves to more environmentally friendly alternatives—is crucial to meet the commitments of the so-called “Green Deal”. The reliance on fossil fuels for heating contributes to environmental degradation. In the provided environmental balance sheet, the “Resources” category is predominant, regardless of the window type. This category, which signifies the depletion of natural resources, is primarily due to the use of fossil fuels as energy sources.

In the era of energy transformation, the presented research results can be used for comparative analyzes of the use of similar windows but different heating systems based on renewable energy sources. Such analyzes will allow for the presentation of measurable environmental benefits resulting from the energy transformation related to the modernization of heating systems in residential buildings. The LCA analysis conducted could be also useful for manufacturers, architects and investors who are looking for solutions that increase the energy efficiency of buildings and reduce their impact on the natural environment. In a later stage of the research, comparative analyzes of the environmental footprint related to the manufacturing and useful life of windows will be carried out, but in the perspective of using renewable energy sources to heat residential buildings.
